# Long-term Outcomes Among Men Undergoing Active Surveillance for Prostate Cancer in Sweden

**DOI:** 10.1001/jamanetworkopen.2022.31015

**Published:** 2022-09-14

**Authors:** Eugenio Ventimiglia, Anna Bill-Axelson, Ola Bratt, Francesco Montorsi, Pär Stattin, Hans Garmo

**Affiliations:** 1 Department of Surgical Sciences, Uppsala University, Uppsala, Sweden; 2 Division of Experimental Oncology/Unit of Urology, Urological Research Institute, IRCCS Ospedale San Raffaele, Milan, Italy; 3 Department of Urology, Institute of Clinical Science, Sahlgrenska Academy, University of Gothenburg, Gothenburg, Sweden; 4 Regional Cancer Centre, Uppsala/Örebro, Uppsala University Hospital, Uppsala, Sweden

## Abstract

**Importance:**

The long-term outcomes among men with prostate cancer (PC) whose disease is managed with active surveillance (AS) remains unknown.

**Objective:**

To develop a simulation model with a 30-year follow-up for men with PC managed with AS.

**Design, Setting, and Participants:**

In this cohort study, a state transition model was created using data from Prostate Cancer data Base Sweden (PCBaSe) on 23 655 men diagnosed with PC and managed with deferred treatment to estimate treatment trajectories. A simulation was performed with 100 000 men in each combination of age at diagnosis, Charlson Comorbidity Index, and PC risk with a follow-up of 30 years.

**Main Outcomes and Measures:**

Death from PC and death from other causes were estimated, and the proportion of time without active PC treatment was assessed until date of death or age 85 years.

**Results:**

This study included 23 655 men from PCBaSe with a median age at diagnosis of 69 years (IQR, 64-74 years). Of these, 16 177 men underwent active surveillance for PC and 7478 underwent watchful waiting. The proportion of men who were diagnosed at age 55 years and died of PC before age 85 years was 9% for very low-risk PC, 13% for low-risk PC, and 15% for intermediate-risk PC. Among men with a Charlson Comorbidity Index of 0 who were diagnosed at age 70 years, the corresponding percentages were 3%, 6%, and 7%, respectively. The mean proportion of remaining life-years without active PC treatment for men diagnosed at age 55 years was 12 of 25 years (48%) for very low-risk PC, 9 of 25 years (36%) for low-risk PC, and 7 of 25 (29%) for intermediate-risk PC. For men aged 70 years, the corresponding numbers were 10 of 13 years (77%), 9 of 13 years (66%), and 8 of 13 years (60%), respectively. Men with intermediate-risk PC who were younger than 60 years at diagnosis had a high risk of PC death (12%-15%) and fewer remaining life-years without active PC treatment (29%-33%). In contrast, men with low-risk PC who were older than 65 years at diagnosis had a lower risk of PC death (3%-5%) and more remaining life-years without active PC treatment (62%-77%).

**Conclusions and Relevance:**

The findings of this Swedish cohort study suggest that active surveillance may be a safe strategy for disease management among men with PC who were older than 65 years at diagnosis.

## Key Points

**Question:**

What are the long-term outcomes among men who have prostate cancer (PC) that is managed with active surveillance?

**Findings:**

In this cohort study of 23 655 men in Sweden, those who were diagnosed with intermediate-risk PC at younger than 60 years had a high risk of PC death and little benefit in terms of time without active PC treatment. In contrast, men older than 65 years with low-risk PC had a lower risk of PC death and longer time without active PC treatment.

**Meaning:**

These findings suggest that active surveillance may be a safe disease management strategy among men older than 65 years with low-risk PC.

## Teaser

This cohort study applies a state transition model to assess 30-year trajectories of prostate cancer (PC) among men in Sweden with low-risk PC or intermediate-risk PC managed with active surveillance.

## Introduction

Active surveillance (AS) is a management strategy for men with low-risk prostate cancer (PC) that entails conversion to active treatment if disease progression occurs, and it is used increasingly worldwide.^1^ The purpose of AS is to minimize the risk of adverse effects of active treatment in men with indolent PC without increasing risk of death.^2^ In Sweden, AS is used increasingly for men with low-risk PC; in 2019, 86% of men diagnosed with low-risk PC in Sweden started AS.^1,3^ However, previous studies on watchful waiting have shown that there is a risk of PC death up to several decades after diagnosis.^4,5^ Because AS did not become commonplace until 2005, there are few data on follow-up beyond 15 years.^6^ Owing to an increased life expectancy, many men on AS will remain at risk of PC death for decades after diagnosis.^7^ Thus, there is a need to assess the long-term outcomes of AS a disease management strategy for PC. Previous studies have reported on the transition from AS to curative treatment and on the risk of PC death for men on AS up to 15 years after starting AS.^8,9,10,11^

To fully assess long-term outcomes among men with PC who are undergoing AS, an extensive PC disease trajectory needs to be investigated given that this trajectory may include several subsequent treatments due to disease progression at several time points.^12^ Because such data are currently not directly available, we created a state transition model that estimated PC disease trajectory, including changes in treatment strategy and outcomes up to 30 years after start of AS.^9,13^ The aim of this study was to apply this model to assess 30-year PC trajectories in men with low- or intermediate-risk PC managed with AS. Specifically, we sought to identify men who would benefit the most from AS in terms of low risk of PC death and of treatment-free years.

## Methods

This cohort study was approved by the Regional Ethical Review Board of Uppsala University. As in all Swedish clinical cancer registers and other quality registers, all men with PC are informed at time of diagnosis and treatment that they will be included in Sweden’s National Prostate Cancer Register (NPCR) unless they opt out; therefore, written informed consent was not required. The study followed the Strengthening the Reporting of Observational Studies in Epidemiology (STROBE) reporting guideline.

### Study Population

We identified men in Prostate Cancer data Base Sweden (PCBaSe) who had been diagnosed at age 40 to 75 years with very low-risk, low-risk, and intermediate-risk PC between 1992 and 2014 according to a modified version of National Comprehensive Cancer Network risk categorization (eTable 1 in the Supplement) and who did not undergo active treatment.^14,15,16^ PCBaSe has been described previously in detail.^17^ In brief, PCBaSe constitutes a linkage between the NPCR and a number of other population-based health care registers and demographic databases.^17^ For this study, we used a subset of PCBaSe containing full disease trajectories.^18^ To assess comorbidity, we used information on discharge diagnoses in the National Patient Registry and the Swedish Cancer Registry at 4-week intervals during follow-up to calculate a time-updated Charlson Comorbidity Index as described previously.^19^ The date for transition from AS to watchful waiting is not recorded in the NPCR (or in any other register in Sweden), so this lack of data was mitigated by using information on dates of prostate biopsy obtained from the National Patient Registry in combination with recordings of watchful waiting and AS in the NPCR as described previously.^9^ In brief, regular use of prostate biopsies was used to model the probability of having received a biopsy, which served as an indication of adherence to AS.

### State Transition Modeling

**Figure 1.**
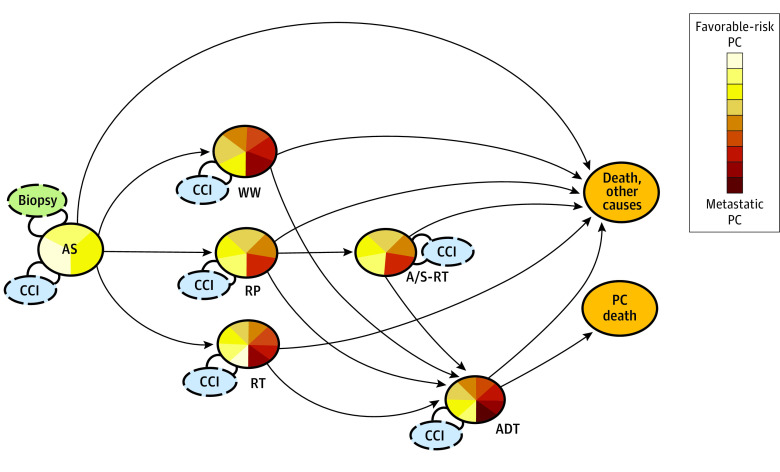
State Transition Model of Transitions Between States Among Men Diagnosed With Prostate Cancer (PC) Managed With Active Surveillance (AS) The states (circles) included were AS, watchful waiting (WW), radical prostatectomy (RP) or radiotherapy (RT), adjuvant or salvage radiotherapy following RP (A/S-RT), androgen deprivation therapy (ADT), PC death, and death from other causes. Arrows indicate transitions. Multicolored circles represent transient states; the size of each colored area represents the proportion of each disease risk category at date of transition. Orange circles represent absorbing states with no further transition possible. Dashed circles represent updated information that affected transition probabilities (eg, biopsy results, Charlson Comorbidity Index [CCI]). The detailed risk categories are provided in eTable 1 in the Supplement.

We used estimates from PCBaSe^Sim^, our previously described simulated state transition model,^13^ to obtain the full PC trajectory including changes of treatment strategy for men managed with AS up to 30 years after diagnosis. The key assumption in our estimates was that after a transition from AS to a new treatment, outcomes after a transition would be similar to those for men who received this treatment as a primary treatment according to the updated disease characteristics at the date of transition. This assumption was based on previous reports that men who underwent radical prostatectomy after an initial period of AS had outcomes similar to those among men who underwent immediate prostatectomy.^20^ The accuracy of this modeling approach was enhanced by including the duration of AS state before a transition and by the estimation of a novel disease risk category once a transition from AS occurred. This model was used previously to describe transitions between treatment states for men with PC via individual-level microsimulation (Figure 1).^13^ In the present study, we prospectively simulated data for 100 000 men for all combinations of age at diagnosis, PC risk category, PSA level, and Charlson Comorbidity Index until age 85 or follow-up of 30 years, whichever occurred first (eTable 1 in the Supplement).

### Transition From Active Surveillance

In brief, each man’s vital status was assessed at 4-week intervals. For a man who was alive at the end of a time step, we determined whether a treatment change had occurred. Such a transition could be direct (eg, when the risk category in the new state was known and remained unchanged) or accompanied by a new risk category (eg, in case of a transition from AS to radical prostatectomy when progression had occurred).^13^

### Statistical Analysis

#### Estimates

The analytical steps in the state transition model have been described previously in detail.^13^ To visualize the timing and occurrence of state transitions in accordance with Figure 1, we calculated time-specific prevalence estimates for men according to age, comorbidity, and PC risk category. To assess the outcome of AS for each of these combinations, we examined the association between PC death and the proportion of life-years after diagnosis without active PC treatment under the assumption that lower risk of PC death and more life-years after diagnosis without active PC treatment represented the greatest benefit of AS.

#### Sensitivity Analysis

Because of the modification in the International Society of Urological Pathology classification of Gleason scoring in 2005, a substantial number of cancers that were graded as Gleason score 6 prior to 2005 would have been graded as Gleason score 7 after 2005.^21^ To compensate for this grade migration, the model was allowed to select a group of men with very low-risk PC with the least favorable prognosis and to upgrade these men to a more advanced risk category. To this end, we identified men in the very low-risk category who had the highest risk of a transition to androgen deprivation therapy and upgraded 5%, 10%, 15%, and 20% of them to a less favorable risk category.

## Results

There were 23 655 men from PCBaSe included in the study with a median age at diagnosis of 69 years (IQR, 64-74 years). Of these, 16 177 underwent AS and 7478 underwent watchful waiting (Table). The median age at diagnosis for men on AS was 67 years (IQR, 62-71 years); a majority of these men (82%) had no comorbidities (Charlson Comorbidity Index = 0). Additionally, most of these men had very low-risk PC (34%) or low-risk PC (59%).

**Table.** Characteristics of Men With Prostate Cancer Registered in Prostate Cancer data Base of Sweden Who Underwent Active Surveillance or Watchful Waiting as Primary Treatment Strategy^a^

Characteristic	Total (N = 23 655)	Active surveillance (n = 16 177)	
Age at diagnosis, median (IQR), y	69 (64-74)	67 (62-71)	
Age group, y			
≤55	1008 (4.3)	809 (5.0)	
56-60	2334 (9.9)	1866 (11.5)	
61-65	4543 (19.2)	3750 (23.2)	
66-70	6172 (26.0)	4948 (30.6)	
71-80	8245 (34.8)	3218 (19.9)	
≥81	1353 (5.7)	1586 (9.8)	
Year of diagnosis			
1992-1997	557 (2.4)	220 (1.4)	
1998-2004	2540 (10.7)	1025 (6.3)	
2005-2008	4542 (19.2)	2388 (14.8)	
2009-2011	7118 (30.1)	5153 (31.8)	
2012-2014	8898 (37.6)	7391 (45.7)	
Tumor stage			
T1a	2557 (10.8)	1288 (8.0)	
T1b	965 (4.1)	364 (2.2)	
T1c	15 248 (64.5)	12 063 (74.6)	
T2	4744 (20.0)	2370 (14.6)	
Missing	141 (0.6)	92 (0.6)	
Nodal stage			
N0	1830 (7.7)	1509 (9.3)	
NX	21 825 (92.3)	14 668 (90.7)	
Gleason score			
Gleason			
6	20 018 (84.6)	14 684 (90.8)	
7 (3 + 4)	2295 (9.7)	1118 (6.9)	
7 (4 + 3)	203 (0.9)	0	
WHO graded	1072 (4.5)	375 (2.3)	
Missing	67 (0.3)	NA	
PSA, ng/mL, median (IQR)^b^	6.0 (4.2-8.2)	5.6 (4.1-7.8)	
Mode of detection			
Screening^c^	10 038 (42.4)	8156 (50.4)	
Lower urinary tract symptoms	7699 (32.6)	4832 (29.9)	
Other symptoms	4265 (18.0)	2387 (14.7)	
Missing	1653 (7.0)	802 (5.0)	
Charlson Comorbidity Index			
0	18 308 (77.4)	13 216 (81.7)	
1	2879 (12.2)	1622 (10.0)	
2	1659 (7.0)	927 (5.7)	
≥3	809 (3.4)	412 (2.5)	
Risk category			
Very low risk	6193 (26.2)	5522 (34.1)	
Low risk	14 881 (62.9)	9501 (58.7)	
Intermediate risk	2581 (10.9)	1154 (7.1)	

Abbreviations: NA, not applicable; PSA, prostate-specific antigen; WHO, World Health Organization.

^a^ Data are presented as No. (%) of patients unless indicated otherwise. Percentages are rounded and therefore may not total 100. The median (IQR) follow-up time was 6.2 (3.7-9.5) years.

^b^ To convert PSA to μg/L, multiply by 1.0.

^c^ Detection via workup after PSA testing in asymptomatic men.

**Figure 2.**
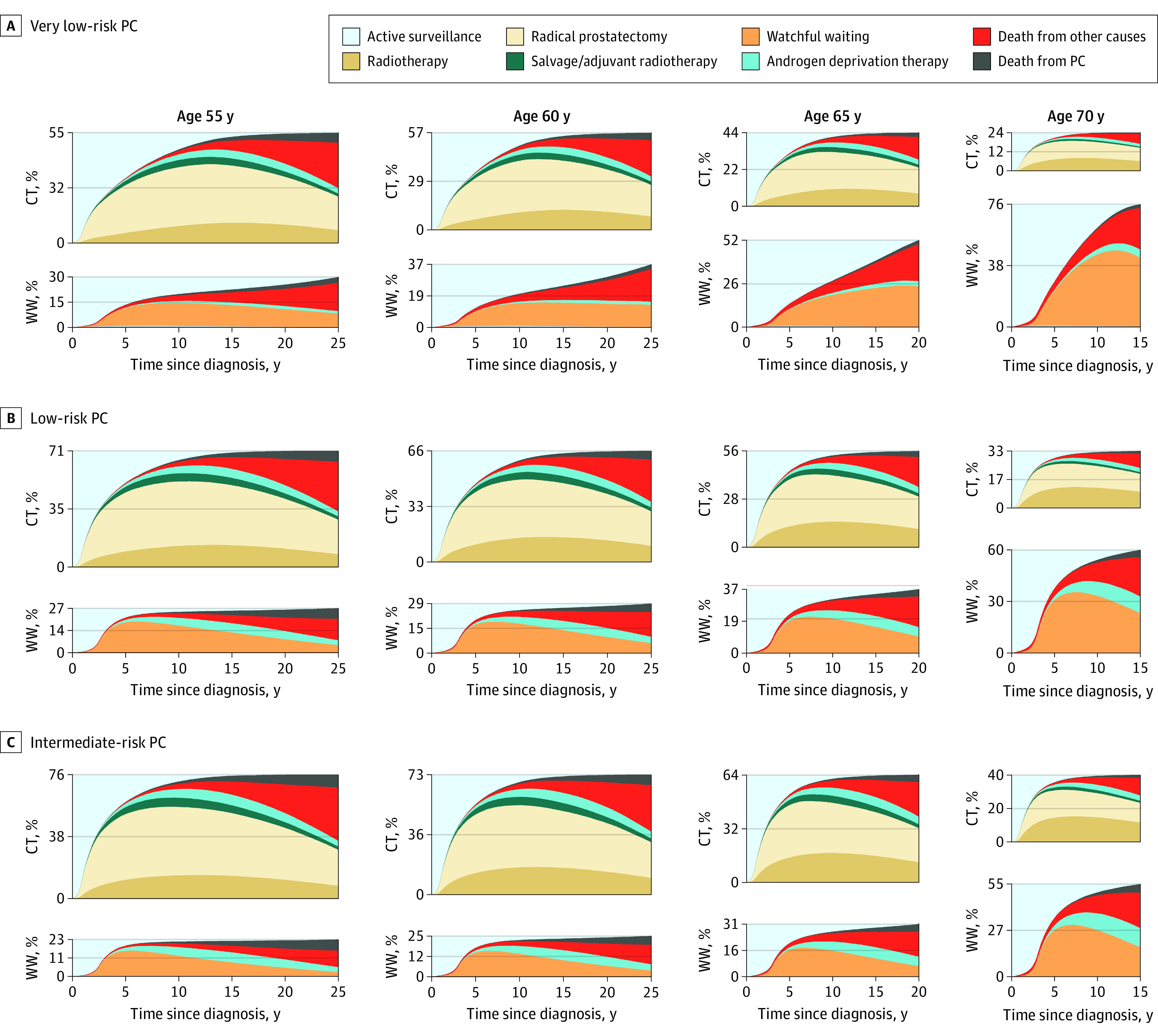
Prevalence of Each State by Prostate Cancer (PC) Risk Category at Each Time Point A, Very low-risk PC. B, Low-risk PC. C, Intermediate-risk PC. Curative treatment (CT) includes radical prostatectomy and radiotherapy. Upper graphs include men who made a transition to CT; lower graphs include men who made a transition to watchful waiting (WW).

Figure 2 shows the time-specific prevalence of each state during follow-up and may be used to estimate the timing and volume of each transition. Transition to radical treatment was much more common in younger men with intermediate-risk PC (eg, 76% in men aged 55 years) than in older men with less aggressive PC (eg, 24% in men aged 70 years with very low-risk PC) (Figure 2). Transition to androgen deprivation therapy after AS or watchful waiting was more common in men aged 70 years with intermediate-risk PC (27%), whereas this transition was observed in only 5% of men aged 55 years with very low-risk PC. The first transitions according to age at diagnosis and PC risk category are detailed in eTable 3 in the Supplement.

**Figure 3.**
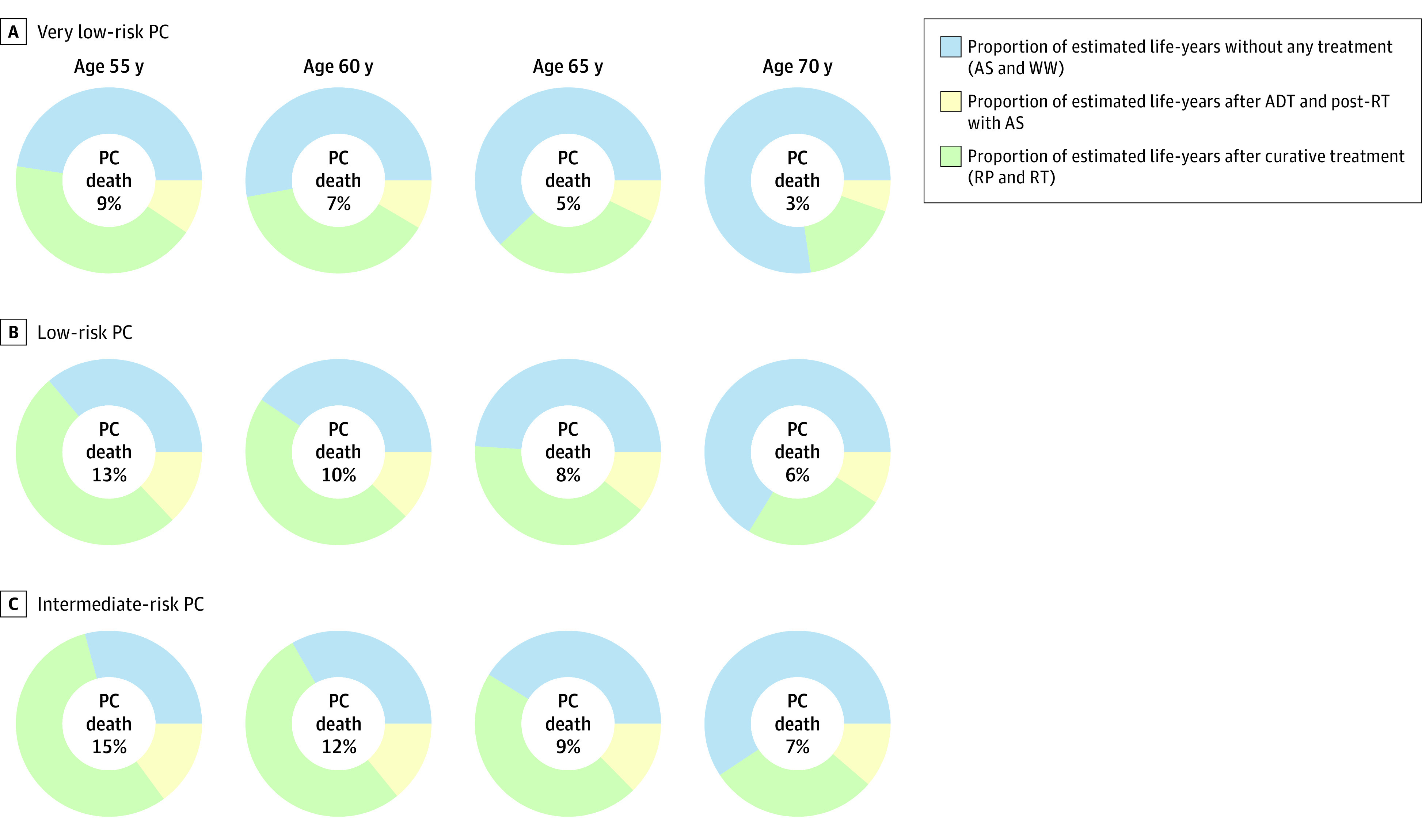
Estimates of Remaining Life-Years Without Active Prostate Cancer (PC) Treatment Based on the results shown in Figure 2, we estimated proportions for each risk category by age group using the ratio between the proportion of the area under the curve for active surveillance (AS) and watchful waiting (WW) and the total remaining life-years. The denominator of these proportions was calculated as life-years resulting from the sum of all areas under the curve in Figure 2 excluding death from PC and death from other causes. ADT indicates androgen deprivation therapy; RP, radical prostatectomy; RT, radiotherapy.

We estimated the remaining life-years spent without active PC treatment as the ratio between the proportion of the area under the curve for AS and watchful waiting (as shown in Figure 2) and the total remaining life-years. A visual representation of these estimates is shown in Figure 3, along with the proportion of PC death for each stratum of age at diagnosis and risk categories.

**Figure 4.**
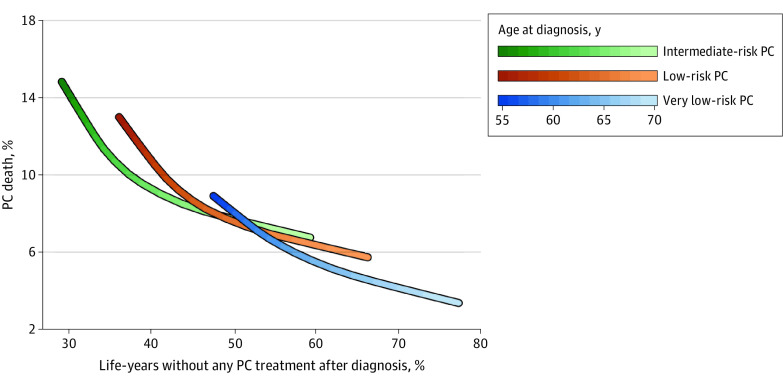
Association Between Proportion of Life-Years Without Active Treatment and Risk of Prostate Cancer (PC) Death

The association between the proportion of time without active treatment and risk of PC death is shown in Figure 4. The proportion of men who had died of PC before age 85 years among men diagnosed at age 55 years vs age 70 years was 9% vs 3% for very low-risk PC, 13% vs 6% for low-risk PC, and 15% vs 7% for intermediate-risk PC, respectively. The mean proportion of remaining life-years without active treatment for men with very low-risk PC was 12 of 25 years (48%) for men diagnosed at age 55 years vs 10 of 13 years (77%) for men diagnosed at age 70 years; for low-risk PC and intermediate-risk PC, the proportions for these age groups were 9 of 25 years (36%) vs 9 of 13 years (66%) and 7 of 25 years (29%) vs 8 of 13 years (60%), respectively.

There was a continuum of an increasing benefit of AS with increasing age at diagnosis and decreasing risk category. Men diagnosed at age 60 years or younger with intermediate-risk PC had fewer life-years without active PC treatment compared with men older than 65 years with very low-risk PC (29%-33% vs 62%-77%, respectively) and a considerably higher risk of PC death (12%-15% vs 3%-5%, respectively). Overall, men older than 65 years with low-risk PC had a high proportion of treatment-free years (53%-70%) and a low risk of PC death (6%-8%). At an earlier time point (15 years), PC death was 2.6% for low-risk PC.

The results of the sensitivity analysis of different risk categories on AS were consistent with the primary results (eTable 2 in the Supplement). In the most favorable scenario (ie, among men with very low-risk PC and excluding 20% of men with the least favorable characteristics), risk of PC death was 3% to 9% for men with very low-risk PC, 5% to 12% for low-risk PC, and 7% to 14% for men with intermediate-risk PC.

## Discussion

In this study based on a nationwide, population-based cohort in Sweden, we modeled outcomes among men with PC who underwent AS that lasted up to 30 years in terms of risk of PC death and remaining life-years without active PC treatment. Old men with low-risk PC had a long time without active treatment and low risk of PC death, whereas young men with intermediate-risk PC had little benefit from AS.

To date, the longest reported follow-up of AS is approximately 15 years, as reported by institutions that pioneered the concept of AS, including the Sunnybrook Active Surveillance program, the Gothenburg screening trial, and the Johns Hopkins University School of Medicine AS cohort.^4,11,14,22,23^ Although these reports have some limitations (eg, relatively small numbers of men followed beyond 10 years and limited follow-up time), they differ from our study in several important ways. First, our simulation was based on population-based data with a capture of 99% of all men diagnosed with PC in the Swedish Cancer Registry to which reporting is mandated by law, whereas the existing series with long-term data began as experimental programs at tertiary referral centers.^4,22,23^ This means that a proportion of men in our cohort would not have been selected for AS at a center of excellence. Moreover, because the follow-up schedule in our study was at the discretion of the treating physician, there is also the issue of difficult standardization of follow-up schemes, and eventually lack of adherence. We believe that the lack of selection and the lack of optimal follow-up are 2 reasons why the observed outcomes on which we base our models are different from those from selected cohorts. Johns Hopkins University School of Medicine, the AS program with the most stringent enrollment criteria,^22^ reports a 15-year cancer-specific survival that is greater than 99%. In this program, most of the enrolled men (>70%) had very low-risk PC and underwent strict follow-up. A scenario more similar to our study has been reported by Godtman et al^11^ and Klotz et al^4^; both series reported a 94% 15-year cancer-specific survival among men with low-risk PC , which is consistent with our model (PC death at 15 years, 2.6%) (Figure 2).

The long-term natural history of currently diagnosed low-risk PC is unknown. Previous studies have shown that risk of PC death persists up to 30 years after diagnosis in men with low-risk PC who undergo watchful waiting.^5,24^ However, in these early studies, the crude classification of cancer stage, tumor grade, and PSA levels makes it difficult to extrapolate these results to men diagnosed and classified with current workup methods.

### Strengths and Limitations

The strengths of our study include the high-quality, population-based data on PC characteristics, treatments, updated information on comorbidity, and a virtually complete nationwide capture that allowed us to model a 30-year follow-up based on a population-based cohort.^17,18^ This model has previously been shown to accurately predict state transitions (ie, changes in treatment strategy in men with PC).^13^ In our model, we updated age, PC characteristics, and comorbidity, which allowed us to estimate transition to watchful waiting as well as the proportion of remaining life-years without treatment until either death or end of follow-up.^12^ In comparison, conventional survival analysis merely provides data on a single end point, neglecting both the path to that specific outcome and time-updated cancer characteristics and comorbidity.^24^

Our study has some limitations. Before 2006, registration in the NPCR did not distinguish between AS and watchful waiting, and prostate volume was not registered in the NPCR until 2008, so we were unable to calculate PSA density for men diagnosed before that time point. Furthermore, the modification of Gleason scoring by the International Society of Urological Pathology in 2005 resulted in tumor grade inflation.^21^ However, our sensitivity analyses confirmed that such limitations did not interfere with our model estimates. There are Swedish guidelines with detailed recommendations for inclusion criteria for AS and instructions for follow-up, and adherence to these guidelines was recently shown to be quite high,^1^ with 92% of Swedish men with very low-risk PC being managed with AS in 2020.^3^ The definitions of AS and watchful waiting in the NPCR are consistent with international guidelines.^6,25,26,27^ Compliance with repeat biopsy has not been very high in Sweden,^28^ although it has improved considerably since the introduction and diffusion of multiparametric prostate magnetic resonance imaging.^29^

To the best of our knowledge, this study is the first to compare the benefit of increased life-years without active treatment (ie, avoiding the high risk of PC death) with the adverse effects of treatment. As expected, we found a greater benefit among older men with low-risk PC vs younger men with intermediate-risk PC. Our results are likely to depict a worst-case scenario given that our model was based on nationwide, population-based quality of care registry data rather than data from tertiary referral centers, and our inclusion period overlapped with the aforementioned limitations in disease classification and indication for deferred treatment. Despite these limitations, our findings could help clinicians and patients with PC select optimal treatment strategies. Additionally, the recent introduction of new diagnostic tools not specifically considered in this study (eg, multiparametric prostate magnetic resonance imaging) may improve the selection process when assigning men to AS, which may lead to better long-term outcomes.

## Conclusions

The findings of this Swedish cohort study suggest that men older than 65 years with low-risk PC had a high proportion of treatment-free years (53%-70%) and a low risk of PC death (6%-8%), hence AS was indicated among men in this subgroup. In contrast, in men younger than 65 years, AS appeared to be indicated only in those with very low-risk PC. Our state transition model based on data for men who were diagnosed between 1992 and 2014 likely provides a worst-case scenario that may improve in the future owing to enhanced diagnostic technology.

## Notes

Supplement. **eTable 1.** Prostate Cancer Risk Categories for Men Included in the Study Sample **eTable 2.** Sensitivity Analysis for Prostate Cancer Death According to the Proportion of Men With Poor Prognosis Very-Low Risk Prostate Cancer Upgraded to Higher Risk Group **eTable 3.** First Transitions for Men on Active Surveillance According to Age at Diagnosis and Prostate Cancer Risk Category
