# Supplementary material for: Long-term Outcomes Among Men Undergoing Active Surveillance for Prostate Cancer in Sweden
Source: JAMA Netw Open. 2022 Sep 14;5(9):e2231015. doi: 10.1001/jamanetworkopen.2022.31015 (PMC9475386; doi:10.1001/jamanetworkopen.2022.31015)
Supplement: Supplement. — eTable 1. Prostate Cancer Risk Categories for Men Included in the Study Sample eTable 2. Sensitivity Analysis for Prostate Cancer Death According to the Proportion of Men With Poor Prognosis Very-Low Risk Prostate Cancer Upgraded to Higher Risk Group eTable 3. First Transitions for Men on Active Surveillance According to Age at Diagnosis and Prostate Cancer Risk Category [file jamanetwopen-e2231015-s001.pdf]

Supplemental Online Content

Ventimiglia E, Bill-Axelson A, Bratt O, Montorsi F, Stattin P, Garmo H. Long-term outcomes among men undergoing active surveillance for prostate cancer in Sweden. *JAMA Netw Open*. 2022;5(9):e2231015. doi:10.1001/jamanetworkopen.2022.31015

eTable 1. Prostate Cancer Risk Categories for Men Included in the Study Sample
eTable 2. Sensitivity Analysis for Prostate Cancer Death According to the Proportion of Men With Poor Prognosis Very-Low Risk Prostate Cancer Upgraded to Higher Risk Group
eTable 3. First Transitions for Men on Active Surveillance According to Age at Diagnosis and Prostate Cancer Risk Category

This supplemental material has been provided by the authors to give readers additional information about their work.

© 2022 Ventimiglia E et al. *JAMA Network Open.*

eTable 1. Prostate Cancer Risk Categories for Men Included in the Study Sample

**PCa risk category**	
Very low-risk	T1c<br>positive cores ≤ 33%<br>cancer length ≤8mm<br>Gleason score 6<br>PSA<10 ng/ml,<br>prostate volume < 90cc<br>>5 core biopsies performed<br>PSA density < 0.15 ng/ml/cc
Low-risk, not very low	Gleason score 6<br>PSA<10 ng/ml,<br>T1 or T2 with at least one of the following characteristics:<br>• prostate volume ≥ 90cc<br>• PSA density ≥ 0.15 ng/ml/cc<br>• ≤5 core biopsies performed<br>• positive cores > 33%<br>• cancer length > 8 mm<br>or GGG 1, T1 or T2 with 10 ng/ml ≤ PSA<15 ng/ml
Intermediate-risk	Gleason score 7 (3+4)<br>PSA<10 ng/ml<br>T1 or T2

© 2022 Ventimiglia E et al. *JAMA Network Open.*

eTable 2. Sensitivity Analysis for Prostate Cancer Death According to the Proportion of Men With Poor Prognosis Very-Low Risk Prostate Cancer Upgraded to Higher Risk Group

Proportion of upgraded men	Very low-risk	Low-risk	Intermediate-risk
0%	3-9%	6-13%	7-15%
5%	3-9%	6-13%	7-15%
10%	3-9%	5-12%	7-15%
15%	3-9%	5-13%	7-14%
20%	3-9%	5-12%	7-14%

© 2022 Ventimiglia E et al. *JAMA Network Open.*

eTable 3. First Transitions for Men on Active Surveillance According to Age at Diagnosis and Prostate Cancer Risk Category

Age at diagnosis	Prostate cancer risk group	RP	RT	WW	ADT	DO
50 years	Very low-risk	47,4%	17,2%	19,3%	1,8%	8,6%
	Low-risk	51,9%	18,8%	23,4%	0,0%	3,6%
	Intermediate-risk	56,3%	20,0%	20,2%	0,0%	2,4%
60 years	Very low-risk	40,4%	16,6%	29,9%	1,7%	11,2%
	Low-risk	45,8%	20,6%	24,0%	0,0%	7,3%
	Intermediate-risk	50,2%	22,7%	20,7%	0,0%	5,1%
65 years	Very low-risk	30,0%	14,2%	44,0%	1,2%	10,6%
	Low-risk	35,9%	20,0%	30,1%	0,0%	12,9%
	Intermediate-risk	40,7%	23,3%	25,4%	0,0%	9,9%
70 years	Very low-risk	13,7%	9,9%	66,5%	1,2%	8,7%
	Low-risk	17,6%	15,8%	55,0%	0,0%	11,6%
	Intermediate-risk	20,4%	19,5%	49,7%	0,0%	10,3%

ADT: androgen deprivation therapy without previous watchful waiting, DO: death from other causes, RP: radical prostatectomy, RT: radiotherapy

© 2022 Ventimiglia E et al. *JAMA Network Open.*
